# Electromyographic analysis of the three subdivisions of gluteus medius during weight-bearing exercises

**DOI:** 10.1186/1758-2555-2-17

**Published:** 2010-07-12

**Authors:** Kieran O'Sullivan, Sharon M Smith, David Sainsbury

**Affiliations:** 1 Physiotherapy Dept, University of Limerick, Ireland

## Abstract

**Background:**

Gluteus medius (GM) dysfunction is associated with many musculoskeletal disorders. Rehabilitation exercises aimed at strengthening GM appear to improve lower limb kinematics and reduce pain. However, there is a lack of evidence to identify which exercises best activate GM. In particular, as GM consists of three distinct subdivisions, it is unclear if GM activation is consistent across these subdivisions during exercise. The aim of this study was to determine the activation of the anterior, middle and posterior subdivisions of GM during weight-bearing exercises.

**Methods:**

A single session, repeated-measures design. The activity of each GM subdivision was measured in 15 pain-free subjects using surface electromyography (sEMG) during three weight-bearing exercises; wall squat (WS), pelvic drop (PD) and wall press (WP). Muscle activity was expressed relative to maximum voluntary isometric contraction (MVIC). Differences in muscle activation were determined using one-way repeated measures ANOVA with post-hoc Bonferroni analysis.

**Results:**

The activation of each GM subdivision during the exercises was significantly different (interaction effect; p < 0.001). There were also significant main effects for muscle subdivision (p < 0.001) and for exercise (p < 0.001). The exercises were progressively more demanding from WS to PD to WP. The exercises caused significantly greater activation of the middle and posterior subdivisions than the anterior subdivision, with the WP significantly increasing the activation of the posterior subdivision (all p < 0.05).

**Discussion:**

Posterior GM displayed higher activation across all three exercises than both anterior and middle GM. The WP produced the highest %MVIC activation for all GM subdivisions, and this was most pronounced for posterior GM. Clinicians may use these results to effectively progress strengthening exercises for GM in the rehabilitation of lower extremity injuries.

## Background

The primary role of gluteus medius (GM) is to stabilise the pelvis and control femoral motion during dynamic lower extremity motion [1-3]. Clinically, dysfunction of GM has been implicated in numerous musculoskeletal disorders including low back pain, patellofemoral pain syndrome and numerous other lower limb injuries [1,4-6]. Addressing dysfunction of hip muscles such as GM can significantly improve lower limb kinematics, assist in injury prevention, improve athletic performance and result in decreased pain [2,5-10].

Gluteus medius attaches to the entire length of the iliac crest, the external ilium between the posterior and anterior gluteal lines, the gluteal fascia, the posterior border of tensor fascia lata (TFL) and the overlying ITB [11,12]. It is a segmented hip muscle consisting of three distinct portions; anterior, middle and posterior [13,14], forming a broad united tendon that wraps around, and inserts onto, the greater trochanter of the femur [15-17]. The more vertical anterior and middle portions of gluteus medius appear better positioned to abduct the hip, than the more horizontal posterior portion [1,9,15,17-20]. There has also been controversy over whether gluteus medius is primarily activated during medial rotation [1] or lateral rotation [21]. Ireland et al. [21] demonstrated significant weakness of hip abduction and lateral rotation in female subjects with patellofemoral pain, when compared to matched controls. This weakness of lateral rotation was attributed to gluteus medius dysfunction. In contrast, Earl [1] observed the highest activation of gluteus medius when combining an abduction and medial rotation task. The orientation and insertion patterns of the anterior and posterior portions appears to reflected their proposed actions as medial and lateral rotators respectively, in line with the findings from electromyography (EMG) studies [1,17,21].

It may be inappropriate to extrapolate the activation of one subdivision of GM to the muscle as a whole [1,21], owing to the functional subdivisions within each muscle. Some clinicians view GM as a homogenous muscle and prescribe common rehabilitation exercises that have been postulated to have a strengthening effect for all of GM [5,20]. Previous studies have mostly analysed the muscle activity of GM as a relatively homogenous muscle during a variety of rehabilitation exercises [9,20,22,23]. Typically these studies only use one electrode to evaluate the effect of these exercises on GM as one large muscle belly. However, there is no current literature to recommend effective strengthening exercises that target each individual subdivision of GM. This is of concern as the one previous study which has examined muscle activity levels in all three GM subdivisions during functional tasks [13] demonstrated that there were significant differences between the amplitude and duration of activation of each subdivision. Another recent study [24] also demonstrated significant differences in the activation of the three GM subdivisions, albeit during non-functional non weight-bearing isometric hip movements. Further research is thus needed to explore the degree of muscle activity for each subdivision during a variety of clinically used strengthening exercises. Greater delineation of which exercises best recruit each GM subdivision is of interest to clinicians as this may help develop more effective exercise prescription and treatment [20,22,23].

Weight-bearing strengthening exercises have been shown to produce significantly higher GM activity in comparison to non-weight-bearing exercises [13,20,25]. This may be related to the need for greater muscular control due to the greater external torque forces on the femur and the pelvis. Three common unilateral, weight-bearing exercises used in clinical practice are the wall squat (WS), pelvic drop (PD) and wall press (WP), possibly reflecting the fact they mimic functional tasks which may be painful or difficult. The WP, which combines frontal and transverse plane loading, is considered to particularly target posterior GM, which is commonly implicated in lower limb injuries [26]. However this has not been investigated in previous studies.

Therefore, the purpose of this study was to investigate the degree of muscle activity in the anterior, middle and posterior subdivisions of GM during these three common exercises; WS, PD and WP. This study also aimed to identify which of the exercises generated the highest muscle activity. It was hypothesised that the WP exercise would demonstrate higher activation levels, particularly in the posterior subdivision due to its proposed role in hip external rotation.

## Methods

This study was approved by the local university research ethics committee.

### Participants

Fifteen healthy subjects (7 male, 8 female) were recruited from within the university campus. Written informed consent was obtained from all subjects prior to testing. Subjects were made aware of their right to withdraw from the study at any time. Subjects were included if they were aged between 18-30 years and had no back or lower limb injury requiring treatment in the past 6 months, similar to previous studies assessing GM activity [1,9,20]. Subjects were also screened using the Modified Physical Activity Readiness Questionnaire in advance of testing [27]. The participants' mean (±SD) age was 22 (±4) years, height was 170 (±12) cm, body mass was 68 (±12) kg, and body mass index was 23 (±3) kg/m^2^.

### Procedures

Subjects attended a one-hour testing session in the university research laboratory. Subjects initially completed a 5-minute aerobic warm-up at a self-selected pace on a treadmill, as well as gentle lower limb stretches to minimise the risk of muscle soreness and muscle fatigue [28].

### Electromyography

Each subject's right leg was tested. After warm-up, the skin was prepared for electrode placement by abrading the skin with fine sandpaper, shaving any hair and cleansing the skin with isopropyl alcohol solution to reduce skin impedance, in line with recommendations [29,30]. A Motion Lab Systems MA-300 multi-channel EMG system (Motion Lab Systems, USA, Inc., Baton Rouge, Louisiana) was used to collect EMG data using bipolar, pre-amplified, circular electrodes which were 144 mm^2 ^in size with a fixed inter-electrode distance of 18 mm. The sample rate was set at 1250 Hz per channel, with a bandwidth of 5-500 Hz, and a gain setting of 2000. The common mode rejection ratio was >100 dB at 60 Hz.

**Figure 1:**
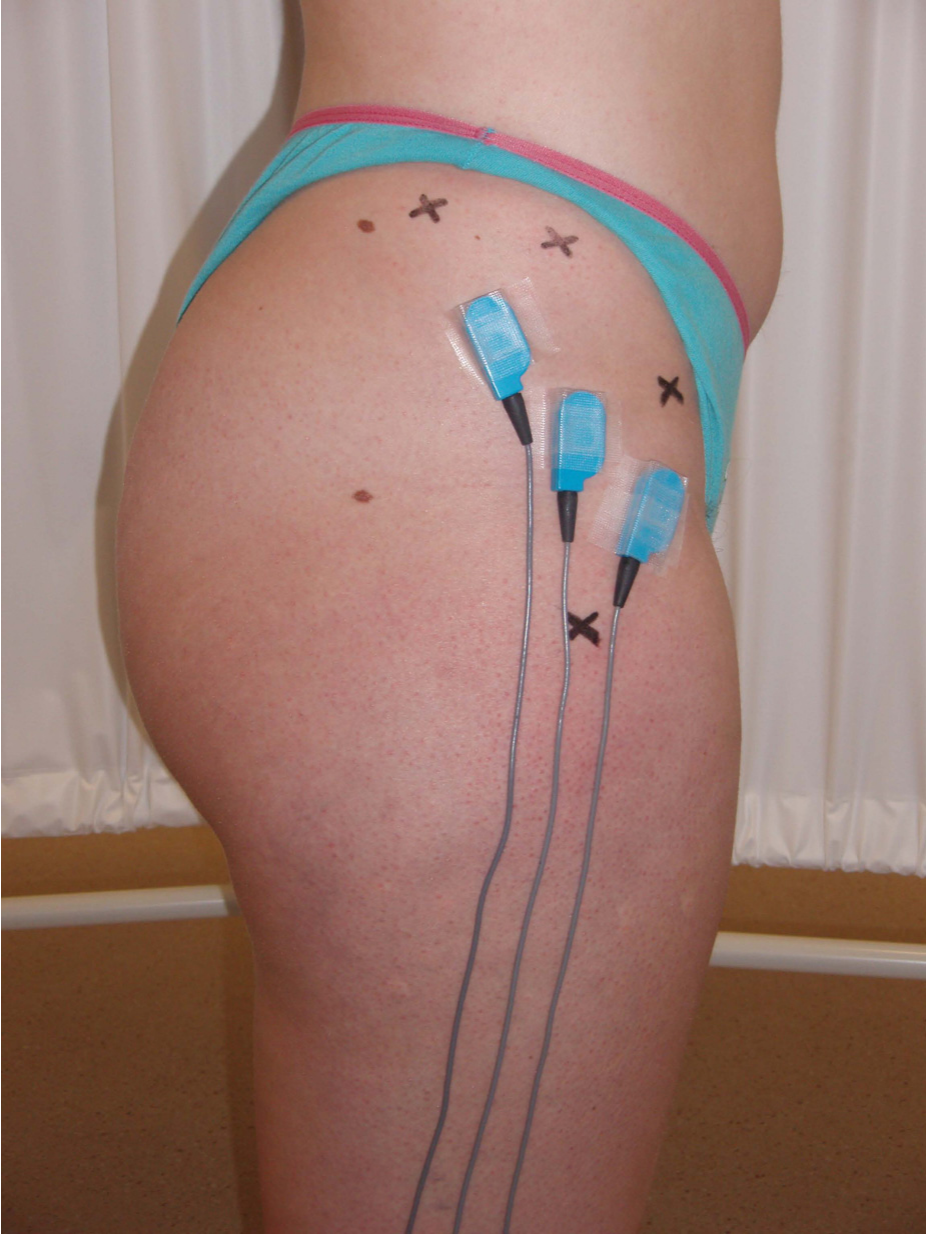
**Electrode placements for the posterior, middle and anterior subdivisions of gluteus medius**. X marks the landmarks use to locate the electrodes; ASIS, iliac crest, greater trochanter, and the posterior ilium. The posterior ilium landmark used was 20% of the distance between the iliac crest and L4-L5 interspace.

SENIAM guidelines [30] describe only one electrode position for GM. Therefore, electrode placement positions for each GM subdivision were modified based on previous EMG studies [1,3] , anatomical dissection studies [15,17,31] and textbook illustrations [11,32]. The anterior GM electrode was placed 50% of the distance between the anterior superior iliac spine (ASIS) and the greater trochanter. The middle GM electrode was placed 50% of the distance between the greater trochanter and the iliac crest, similar to previous research [1,3]. The posterior GM electrode was placed 33% of the distance between the posterior ilium and the greater trochanter (Figure 1). The posterior ilium landmark used was 20% of the distance between the iliac crest and L4-L5 interspace. Correct location of the electrodes was visually confirmed by examining the EMG output while applying manual resistance to hip abduction [11]. Electrodes were checked for good contact prior to all exercises [33]. A small anatomical dissection study and a preliminary pilot study using real-time ultrasound confirmed that GM was the muscle immediately beneath these electrode placements, and not other superficial muscles such as gluteus maximus posteriorly or tensor fascia lata anteriorly. These electrode placements for GM subdivisions are also consistent with a recent paper examining the activation of GM subdivisions during isometric hip contractions [24]. Anatomical landmarks were marked on subjects, and confirmed by a second tester to improve reliability, using a hypoallergenic marker. A reference electrode was placed on the ulnar styloid process [29]. One electrode was placed on each muscle subdivision (anterior, middle and posterior) and orientated parallel to the muscle fibre direction of the individual muscle subdivision [29,34].

EMG data were normalised to maximum voluntary isometric contraction (MVIC), as this is the most reliable method for determining differences in muscle activation during hip abduction exercises in asymptomatic subjects [35]. Many previous studies have simply used abduction as a suitable action for determining MVIC [20,23]. However, since GM acts to rotate as well as abduct [1] it was decided to also assess EMG activity during maximal isometric internal and external rotation, and use the highest EMG reading from all three hip movements to calculate MVIC. This is similar to the standard use of multiple exercises to normalise trunk muscle activation in other studies [36].

MVIC testing was performed using the Biodex Isokinetic Dynamometer, which has been shown to provide reliable and valid measures for torque [37]. Hip abduction was tested in standing with the hip in 30° abduction. Subjects maintained an upright trunk position, with the hip in a neutral flexion/extension and neutral internal/external rotation position, and pushed their leg directly laterally during abduction testing. Internal and external rotations were tested in prone with the hip in neutral rotation and the knee flexed to 90°. The dynamometer resistance pad was placed 2 cm superior to the superior pole of the patella during abduction, and 2 cm superior to the lateral malleolus for internal/external rotation. Prior to testing in each position, three sub-maximal and one maximal contraction were performed for familiarisation purposes and to ensure correct performance [38]. Subjects then performed three MVIC's each of five seconds duration in each direction to allow for normalisation of data, similar to previous trials [9,23]. Standardised verbal encouragement was given to each subject, as this can affect isokinetic output [39]. Subjects were given a 30 second rest period between MVIC trials. The highest muscle activation value for each GM subdivision from any hip contraction direction was recorded, and data obtained from each subsequent weight-bearing exercise trial was then expressed as a percentage of this MVIC.

### Weight-Bearing Exercises

**Figure 2:**
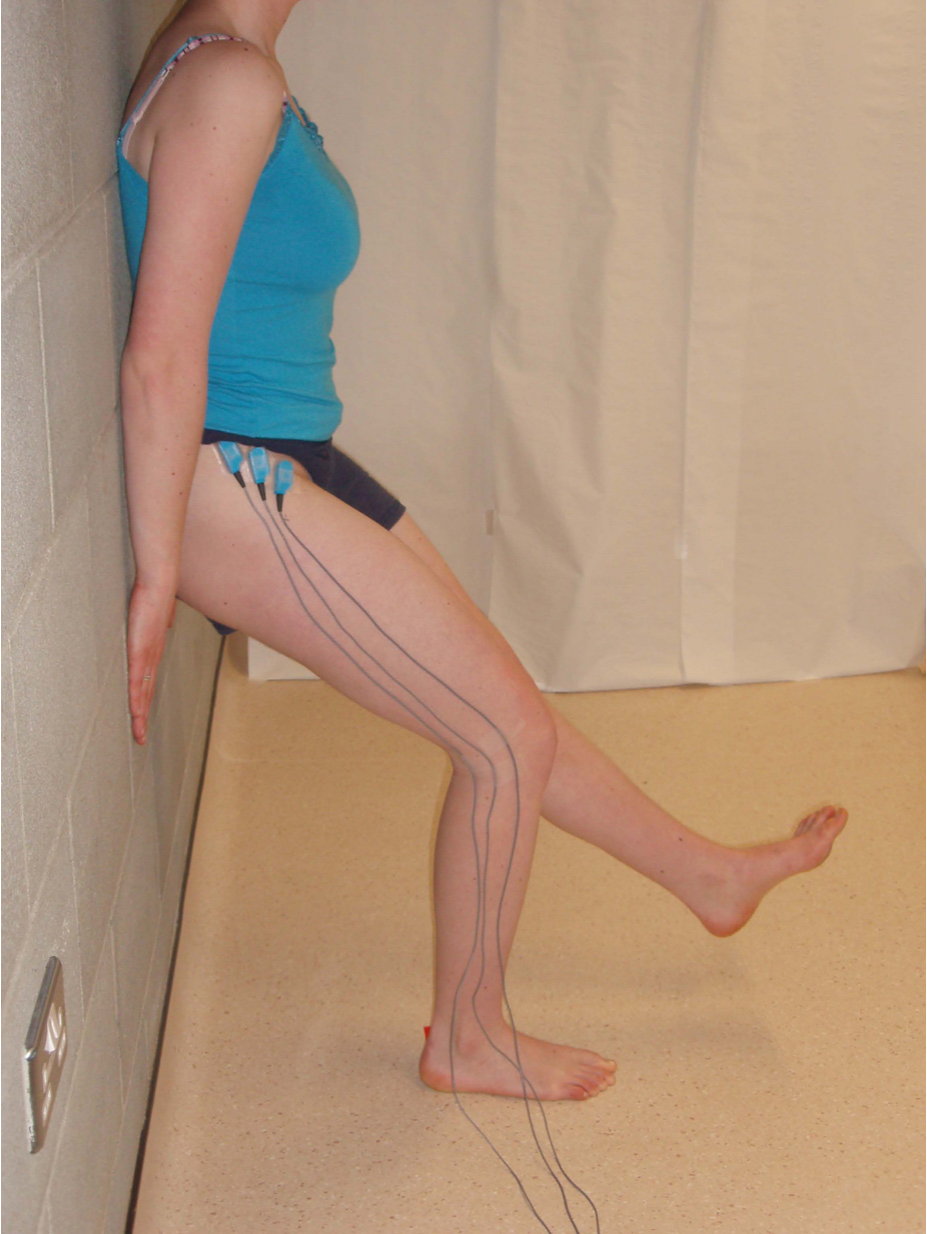
**Subject performing the wall squat (WS) exercise**.

Three variations of unilateral weight-bearing exercises were performed; the unilateral WS, PD and WP. EMG activity was recorded from the supporting lower extremity during each exercise. For the WS exercise subjects stood with their back resting against the wall, heels 30.48 cm from the wall, with their leg perpendicular to the floor [23]. Subjects were asked to maintain a static single leg WS with their right leg for five seconds. Subjects were allowed to lightly touch the wall with their hands in order to maintain their balance (Figure 2).

**Figure 3:**
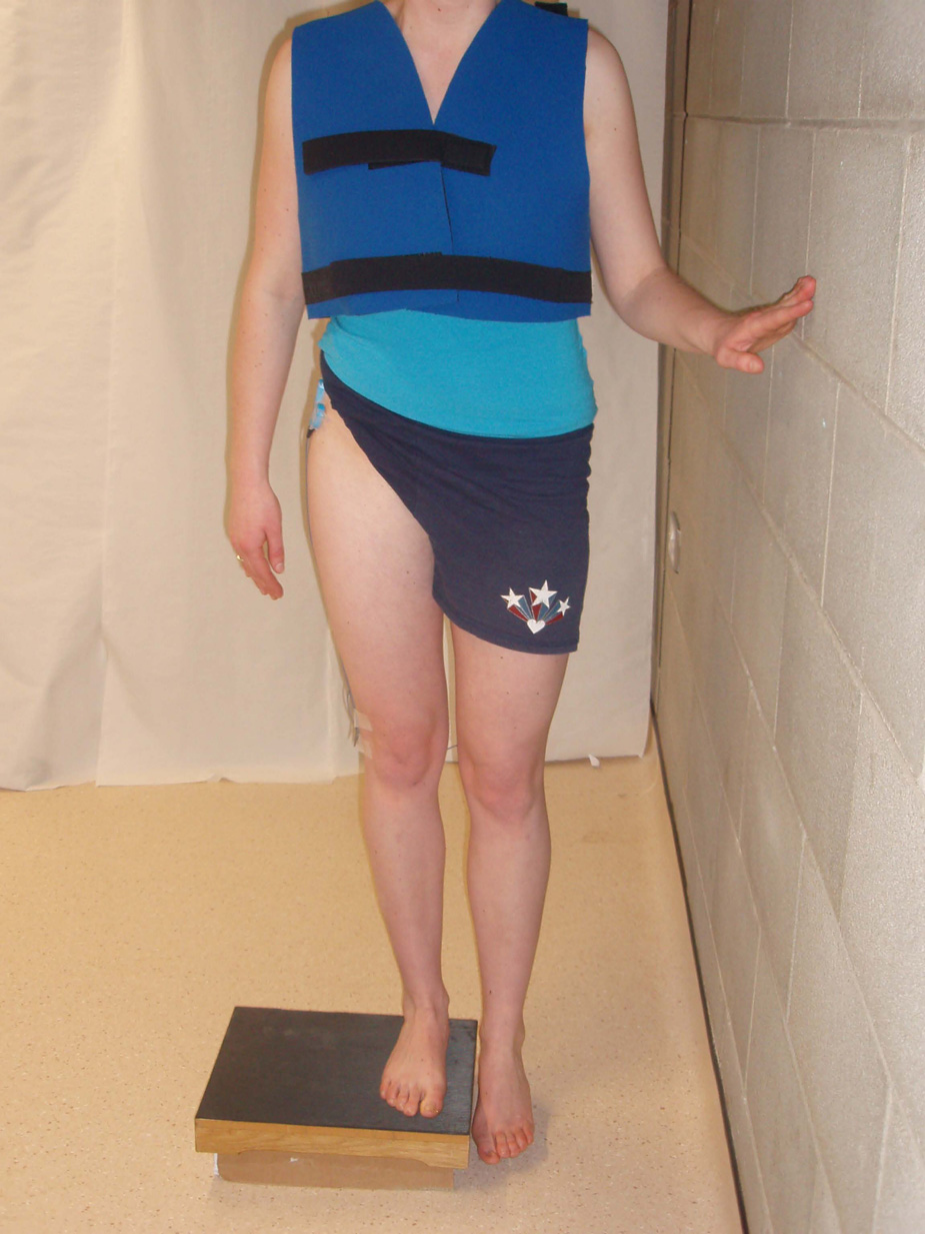
**Subject performing the pelvic drop (PD) exercise**.

For the PD exercise subjects were permitted to lightly touch the wall with one hand, to maintain their balance where necessary [23]. During this exercise, for ease of movement, subjects wore a light velcro jacket to which the backpack and electrodes were connected. Subjects stood on their right lower extremity on a 15 cm step. While maintaining extension of both knees, subjects were asked to lower the left foot toward the floor and then return the foot back to the step [20] (Figure 3). Subjects lightly tipped their first toe off the ground to ensure standardisation and consistency between subjects and to ensure adequate depth of the exercise was achieved each time. This exercise was timed, so that the descent and ascent phases both lasted two seconds.

**Figure 4:**
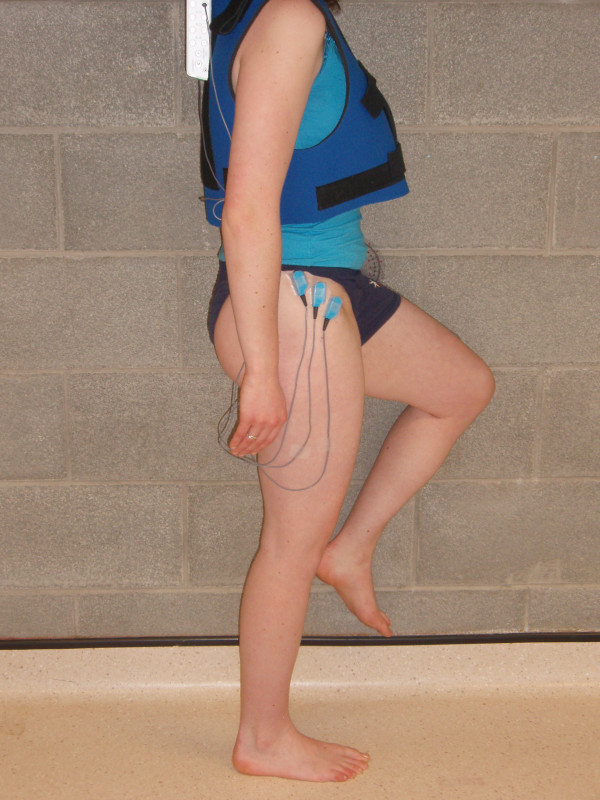
**Subject performing the wall press (WP) exercise**.

During the WP exercise subjects also wore the velcro jacket for ease of movement. Subjects were asked to stand next to a wall with the right limb furthest from the wall. They were then asked to assume a single leg stance position by flexing their left hip to 60 degrees and their left knee to 90 degrees, using goniometric measures. The medial aspect of the right foot was positioned 20 cm from the wall (Figure 4). Subjects were asked to maintain this position while concurrently maximally pushing their left knee, leg and ankle against the wall. They were not specifically asked to contract their right hip muscles. Subjects kept their trunk in a vertical alignment and their pelvis level throughout the exercise [5]. Subjects maintained this isometric contraction for five seconds during each trial. Prior to testing subjects were given three practice trials of each exercise for familiarisation purposes during which any subject performance errors, including pelvic rotation or tilting, were corrected. Subjects performed three repetitions of each exercise, with a 30 second rest period between trials and a one minute rest period between exercises to reduce the possibility of fatigue [9]. The order of exercises was randomised. During data collection, EMG signals were monitored on the computer screen. EMG data were analysed over the entire five second period for all MVIC contractions, as well as for the WS and WP exercises. For the PD exercise, the entire four seconds was analysed with no differentiation between the concentric and eccentric components, as patients normally complete both components together as part of their rehabilitation programme. The EMG data were then full-wave rectified and processed using a root-mean-square (RMS) algorithm over 150 milliseconds [40]. The mean RMS amplitude for each subdivision over the three trials was then calculated, and the average expressed as a percentage of MVIC [20].

### Statistical Analysis

Statistical analysis was performed using SPSS 15.0. Data were normally distributed (Kolmogorov Smirnov, p > 0.05). A one-way repeated measures ANOVA (with post-hoc Bonferroni) was initially performed to determine if any significant differences existed with respect to: (1) subdivision activity, (2) exercise condition and (3) exercise and subdivision interaction. If a significant interaction was present, then pairwise post-hoc comparisons were performed to test for differences between each muscle subdivision and each exercise, similar to previous research [23]. All p-values for pairwise statistical tests were reported after adjusting (Bonferroni) for multiple comparisons, to reduce the risk of a type 1 error. For all statistical tests the alpha level was set at p < 0.05.

## Results

**Figure 5:**
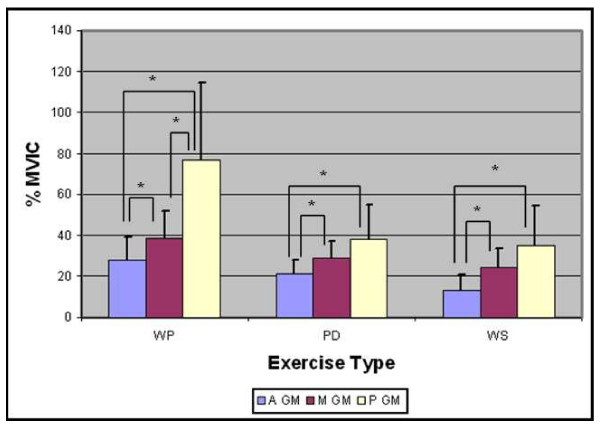
**Mean (+SD) RMS muscle activity for each gluteus medius subdivision (anterior, middle and posterior) during the three weight-bearing exercises (WP, PD, WS)**. Muscle activity expressed as %MVIC.

All 15 subjects completed the test protocol. The mean RMS amplitude of the three subdivisions of GM during the three exercises is displayed in Figure 5. There was a significant interaction between muscle subdivision and exercise type (F_1,28 _= 6.25, p < 0.001). This indicates that the activation of the three subdivisions of GM was significantly different, depending on which exercise was performed. Furthermore, there was a significant main effect for muscle subdivision (F_1,28 _= 21.85, p < 0.001) and exercise (F_1,28 _= 30.35, p < 0.001), indicating that there were significant differences between the muscle subdivisions and between the exercises. Table 1 illustrates the activation of each GM subdivision during each exercise, expressed relative to %MVIC. Post-hoc pairwise comparisons were then used to test the differences between muscle subdivisions, and between exercises.

**Table 1:** Mean (±SD) RMS muscle activity for each gluteus medius subdivision (anterior, middle and posterior) during the three weight-bearing exercises (WP, PD, WS)

	WP	PD	WS
Anterior	27.64 (±11.14)	21.12 (±6.80)	13.30 (±7.50)
Middle	38.60 (±13.22)	28.45 (±8.49)	24.60 (±8.89)
Posterior	76.42 (±38.31)	38.17 (±16.76)	34.82 (±19.86)

Muscle activity expressed as %MVIC.

On examining the significant differences between muscle subdivisions, the mean activation for anterior GM ranged from 13% to 28% MVIC. The anterior subdivision was significantly more active during both the WP (p = 0.001) and PD (p = 0.023) than during the WS, while there was no significant difference in activation between the WP and PD (p = 0.079). For middle GM, the mean activation ranged from 24% to 38% MVIC. The middle subdivision was significantly more active during the WP than either the WS (p = 0.005) or PD (p = 0.027), however there was no significant difference in activation between the PD and WS (p = 0.585). Finally, for posterior GM, the mean activation ranged from 34% to 76% MVIC. The posterior subdivision was significantly more active during the WP than either the WS (p = 0.003) or PD (p = 0.004), while there was no significant difference in activation between the WS and PD (p = 1.0).

On examining the significant differences between exercises, the WS activated the posterior (p = 0.01) and middle (p < 0.001) subdivisions significantly more than the anterior subdivision, however there was no significant difference between the posterior and middle subdivisions during the WS (p = 0.30). The PD activated the posterior (p = 0.004) and middle (p= 0.020) subdivisions significantly more than the anterior subdivision, however there was no significant difference between the posterior and middle subdivisions during the PD (p = 0.052). The WP activated the posterior subdivision significantly more than the anterior (p = 0.001) and middle p = 0.003) subdivisions, and the middle was significantly more active than the anterior subdivision during the WP (p = 0.021).

## Discussion

This is the first study to evaluate the activation of all three subdivisions of GM during weight-bearing exercises. The findings reveal that activation levels of GM varied significantly across each of the subdivisions. Overall, the exercises caused greater activation of the middle and posterior subdivisions than the anterior subdivision, with the WP particularly increasing the activation of the posterior subdivision. Across all muscle subdivisions, the exercises were progressively more demanding from WS to PD to WP, with the WP being particularly effective for posterior GM. Therefore, the results of this study further support the hypothesis [14,15] that there are different functional subdivisions within GM. These results support the findings of Soderberg and Dostal [13] who also found significant variations in EMG activity in each subdivision of GM using fine wire electrodes during a variety of functional tasks. Unfortunately, it is difficult to compare these results with those of Soderberg and Dostal [13] as they analysed the amount of activity in qualitative terms only. Similarly, the findings are consistent with O'Dwyer et al [24] who demonstrated significant differences between GM subdivisions during isometric hip contractions. These results also support the contention that using a single electrode to assess the function of GM may be inappropriate, due to the differences in muscle activation levels identified between GM subdivisions.

The WP exercise generated the highest EMG amplitudes in all three subdivisions. This may relate to the fact that subjects are fully weight-bearing during the WP exercise, whereas subjects supported themselves against the wall during the WS exercise. Furthermore, the WP elicits a considerable rotary force through the hip, unlike the PD or WS exercises, as the force exerted against the wall tends to cause hip internal rotation on the weight-bearing leg. This acts to increase the hip external rotation force required to maintain pelvic and hip posture. These results are consistent with the significant increase in anterior GM activity observed by Earl [1] during an alternative GM weight-bearing exercise requiring a hip internal rotation force to maintain pelvic and hip posture. Despite being used in clinical practice, the effectiveness of the WP in activating GM does not appear to have been evaluated previously. These results suggest that the WP exercise is an effective isometric strengthening exercise for GM, and particularly posterior GM.

Direct comparison with previous research studies is difficult as many only used one surface electrode for GM, although most appear to have used an electrode position similar to the middle GM position in this study. Interestingly, those who have previously examined the WS [23] and PD [20] exercises reported higher %MVIC values for these exercises in their studies. The authors of the current study believe this relates to differences in the study protocol between these other studies and this study. The more demanding normalisation protocol chosen for MVIC testing in the current study, where the highest EMG value obtained in any normalising direction was chosen, may partly explain the relatively lower normalised values obtained during performance of the exercises. The WS was analysed as an isometric exercise, which may explain the %MVIC value of 24% obtained, whereas Ayotte et al. [23] obtained a higher %MVIC value of 52% for a concentric WS. Furthermore, Bolgla and UhI [20] analysed the PD exercise over a shorter two second period, however the PD was analysed over a four second period in the current study as it helped subjects perform the exercise in a smooth manner during pilot testing. The faster PD used by Bolgla and Uhl [20] may explain their %MVIC value of 57%, as opposed to 28% for middle GM in the current study. While the actual %MVIC value obtained with each exercise is not critical, EMG amplitudes can provide clinicians with a guide as to how difficult an exercise is, and how best to progress a patient's rehabilitation program depending on their functional level. EMG amplitudes of greater than 40-60%MVIC have been suggested to provide sufficient stimulus to strengthen muscles [9,41]. Therefore, these findings suggest that only the WP exercise for the posterior GM subdivision elicited sufficient EMG amplitudes to have a strengthening effect. On the other hand, the WS and the PD exercises were shown to produce much lower levels of muscle activation. The fact that previous studies [20,23] obtained higher %MVIC values using these same exercises may lead to clinicians recommending these exercises to strengthen GM. While the WS and PD exercises may be appropriate and effective during rehabilitation, they may be most useful in the early stages in a deconditioned athlete to improve endurance, stability and motor control.

### Clinical Implications

Distinct subdivisions appear to exist within numerous skeletal muscles [42-44]. This study confirms the presence of similar subdivisions within GM, as suggested by anatomical studies [15,17,31]. The presence of these subdivisions may require consideration in clinical assessment as well as rehabilitation. Our results suggest that these GM subdivisions do not work in the exact same manner. However, there is a degree of consistency in the manner in which GM subdivisions are activated by the three exercises. This is consistent with previous suggestions that the gluteal muscles may work together synergistically, according to the load placed on the body, rather than in isolation [14]. Of particular relevance are the recent findings of Cowan et al [43]. Their study [43] demonstrated delayed activation of both anterior and posterior GM in subjects with patellofemoral pain. This further supports the hypothesis that dysfunction of GM is not isolated to one particular subdivision [14]. There is considerable evidence of deficits in hip muscle function in subjects with numerous musculoskeletal disorders [2,23,45-48]. There is also evidence that rehabilitation programmes aimed at increasing the strength and activation of hip muscles such as GM are effective in reducing pain and disability, and improving lower limb kinematics and athletic performance [2,5,46]. The results of this study suggest that the WP is an appropriate exercise if the aim is to activate GM, in particular the posterior subdivision, and may be worth considering as part of GM rehabilitation. This should be considered along with existing research regarding progression of GM rehabilitation [5,49], according to the needs of the individual subject. It is important that future studies evaluate the activation of GM subdivisions in numerous lower limb disorders [43].

### Limitations

The sample size of this study (n = 15) was small, and consisted of young asymptomatic subjects, however this is comparable to previous EMG studies [1,50]. Using surface electrodes always involves a risk that "crosstalk" from nearby muscles, or even adjacent muscle subdivisions, could affect the results [33]. This limitation applies to all sEMG studies, and was minimised by using a small inter-electrode distance as recommended [29]. The optimal electrode placement location for GM subdivisions is unknown, and the electrode placement chosen was based on previous dissection studies [15,17,31] and pilot ultrasound testing. It has also recently been used in the examination of GM activation during isometric hip activation [24]. There remains a possibility that the electrodes were not optimally placed which may have affected the EMG signal [51]. Of additional concern is the fact that part of posterior GM lies deep to the gluteus maximus [17] and hence was inaccessible with sEMG. Therefore, the posterior GM position described reflects the superficial, and not the deep inferior, part of posterior GM. Further research examining the deep inferior portion of posterior GM is required to confirm that these initial findings reflect the activation of the deep posterior GM, which may be different. Indeed, further research examining this using fine-wire EMG is planned. This study examined solely the activation of GM, and not other key muscles involved in movement and stability of the hip [17]. Concurrent recording of the activation of these other muscles would provide a more comprehensive analysis of muscle activity during hip abduction and rotation, and is worthy of further study. The test protocol involved only three exercises, and clearly other movement patterns, and other body positions, are worthy of consideration. The lack of a standardised position for generating a true MVIC of GM limits comparisons of results to other studies, as highlighted earlier. Despite this, the within-subject design allows comparison of the varying demands between exercises within this study. It is important to remember that these results are expressed as %MVIC for each individual subdivision, and are not the actual raw EMG activation of each subdivision. For example, the posterior subdivision of GM actually had the lowest mean RMS activity during MVIC testing. In addition, during the exercises the RMS amplitude of the posterior subdivision was regularly not the highest observed. However, when expressed as %MVIC, the posterior subdivision was working closer to its' maximum level than the anterior or middle subdivisions. It is important to be aware of this potential confusion, so as not to interpret the results as demonstrating that the posterior subdivision had the highest level of GM activation in general, which it clearly did not. This is consistent with research indicating that the primary action of GM is abduction and internal rotation [1,3]. A larger amount of subcutaneous adipose tissue under the posterior GM electrode may explain the decreased raw sEMG signal in part, but each exercise was then normalised to %MVIC to control for this. This study examined only muscle activation amplitude, and not timing, which is worthy of future study as it may be important in numerous musculoskeletal disorders [43,46,48,52]. Hip and knee angles during the WS, and the force exerted against the wall by subjects during the WP, were not standardised, which could significantly influence results. Subjects were not asked to isometrically contract their right hip muscles during the WP, which could have resulted in even higher levels of muscle activation. The PD exercise was not separated into concentric and eccentric components to reflect performance of the exercise in clinical rehabilitation, although higher EMG activity during the concentric period was noticed, similar to previous research [53]. Despite these limitations, this study remains the first to evaluate the muscle activity in all three GM subdivisions during weight-bearing rehabilitation exercises. The results may help clarify some existing confusion in the literature, and guide both clinical practice and future studies on clinical populations.

## Conclusion

GM activation was not consistent across the three GM subdivisions during the three exercises analysed. The WP exercise produced the highest activation levels in all three GM subdivisions, and appears to provide an adequate stimulus for strengthening posterior GM. However, further studies using fine-wire emg in a large sample of symptomatic individuals are required to clarify these initial findings.

## Competing interests

The authors declare that they have no competing interests.

## Authors' contributions

KOS and DS were involved in conception and design of the study, data analysis and interpretation, as well as drafting and editing the final document for publication. SS was involved in conception and design of the study, data collection, data analysis and interpretation, as well as drafting and editing the final document for publication.
